# Supplementary material for: Dopamine D2 Receptor Agonist Binding Kinetics—Role of a Conserved Serine Residue
Source: Int J Mol Sci. 2021 Apr 15;22(8):4078. doi: 10.3390/ijms22084078 (PMC8071183; doi:10.3390/ijms22084078)
Supplement: Supplementary file 1 [file ijms-22-04078-s001.zip › supplementary table S1.pdf]

International Journal of
Molecular Sciences

MDPI

Supplementary material Ågren et al.

Supplementary Table S1. Estimated reverse (k_{off}) rate constants at WT D_2R when agonist availability was terminated by haloperidol application instead of agonist washout.

**Agonist**	**k_{off} ± SEM (s^{-1} × M^{-1})**	**N**
DA	0.223 ± 0.032	3
(S)-5-OH-DPAT	0.027 ± 0.002	4
(R)-5-OH-DPAT	0.033 ± 0.005	3
