# Supplementary figures and images for: Dopamine D2 Receptor Agonist Binding Kinetics—Role of a Conserved Serine Residue
Source: Int J Mol Sci. 2021 Apr 15;22(8):4078. doi: 10.3390/ijms22084078 (PMC8071183; doi:10.3390/ijms22084078)

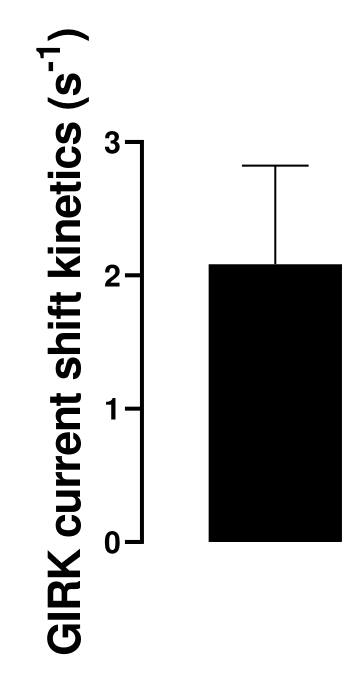

Supplement: Supplementary file 1 [file ijms-22-04078-s001.zip › Supplementary Fig S3 IJMS R1.jpg]

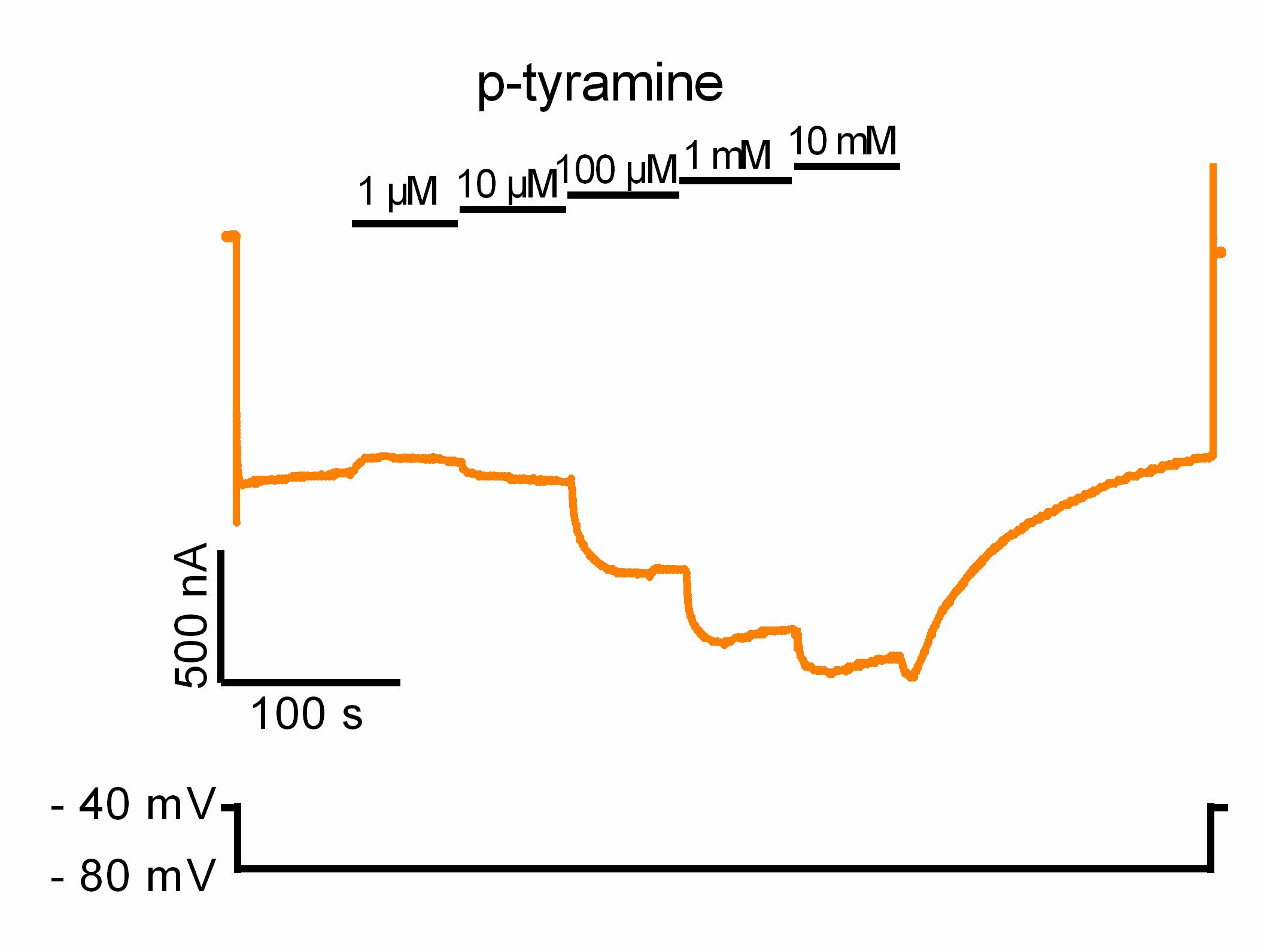

Supplement: Supplementary file 1 [file ijms-22-04078-s001.zip › Supplementary Figure S1 IJMS R1.jpg]

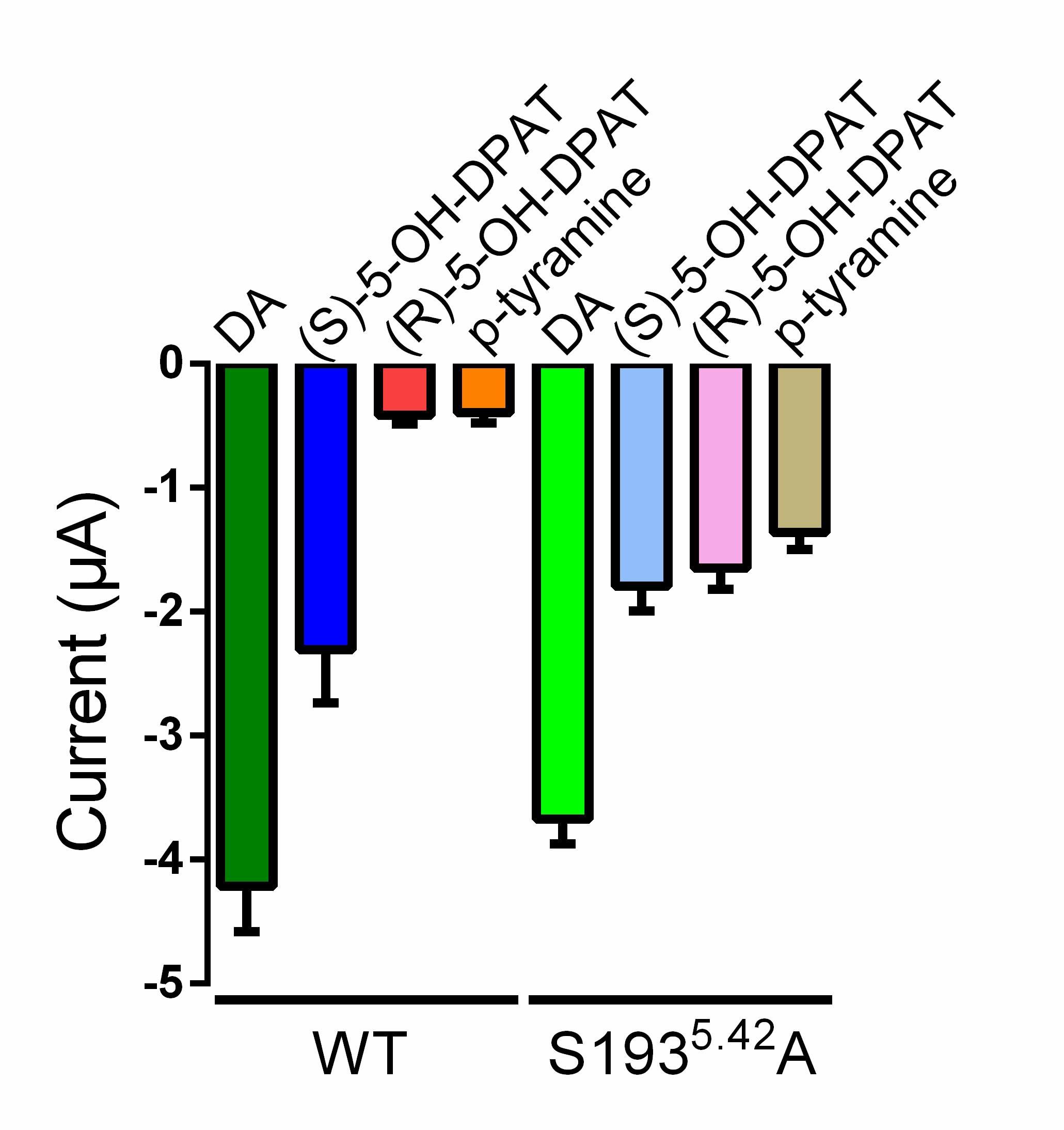

Supplement: Supplementary file 1 [file ijms-22-04078-s001.zip › Supplementary Figure S2 IJMS R1.jpg]

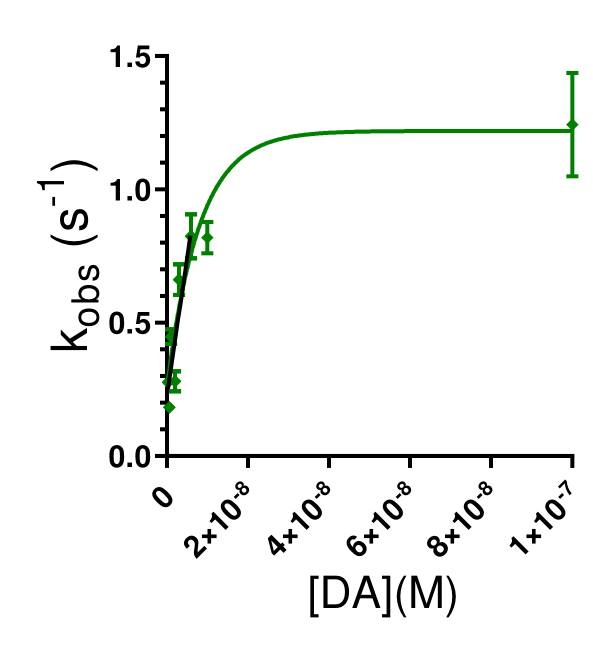

Supplement: Supplementary file 1 [file ijms-22-04078-s001.zip › Supplementary Figure S4 IJMS R1.jpg]
